# Pseudoactinomycotic Radiate Granules in the Gynecological Tract: A Case Report

**DOI:** 10.7759/cureus.69062

**Published:** 2024-09-10

**Authors:** Adaugo Nwanguma, Kshitij Arora

**Affiliations:** 1 Pathology and Translational Pathobiology, Louisiana State University Health Sciences Center, Shreveport, USA

**Keywords:** actinomycotic granules, biopsy, endometrium, pelvic inflammatory disease, pseudoactinomycotic granules

## Abstract

Actinomyces can cause severe infections in the gynecological tract, such as pelvic inflammatory disease (PID) and tubo-ovarian abscess. It's essential to accurately diagnose actinomycotic granules (AMGs) in gynecological specimens to ensure proper treatment, significantly differentiating them from pseudoactinomycotic radiate granules (PAMRAGs), a non-pathologic condition. This article describes a case of a 61-year-old postmenopausal woman with an intrauterine device (IUD) who was diagnosed with PAMRAGs in an endometrial biopsy specimen. This case highlights the challenges in diagnosis, emphasizing the need to understand the distinguishing features and staining properties of PAMRAGs and AMGs to avoid diagnostic errors and awareness of the histological distinguishing features and staining properties of PAMRAGs and AMGs to avert diagnostic mistakes.

## Introduction

Pseudoactinomycotic radiate granules (PAMRAGs) are non-pathogenic findings that can create a diagnostic challenge, as they closely resemble actinomycotic granules (AMGs). Actinomyces are filamentous Gram-positive anaerobic bacteria commonly found in the oral, reproductive, and gastrointestinal tract [1-4]. However, unlike PAMRAGs, actinomyces species may be associated with pelvic inflammatory disease (PID) and tubo-ovarian abscess in patients with long-term intrauterine devices (IUD). Correctly identifying AMGs on histology demands antibiotic treatment and follow-up, while PAMRAGs are common among IUD and non-IUD users and do not require antibiotic treatment. Hence, distinguishing between these conditions is crucial in histology H&E and ancillary stains. Several instances of both conditions coexisting warrant a precise morphological diagnosis of both lesions. We report the case of a 61-year-old lady with an IUD who presented with endometrial biopsy findings of PAMRAGs. This case report aims to emphasize the morphological distinguishing features of PAMRAGs and AMGs to assist in diagnosis and treatment, with a review of relevant literature.

## Case presentation

**Figure 1:**
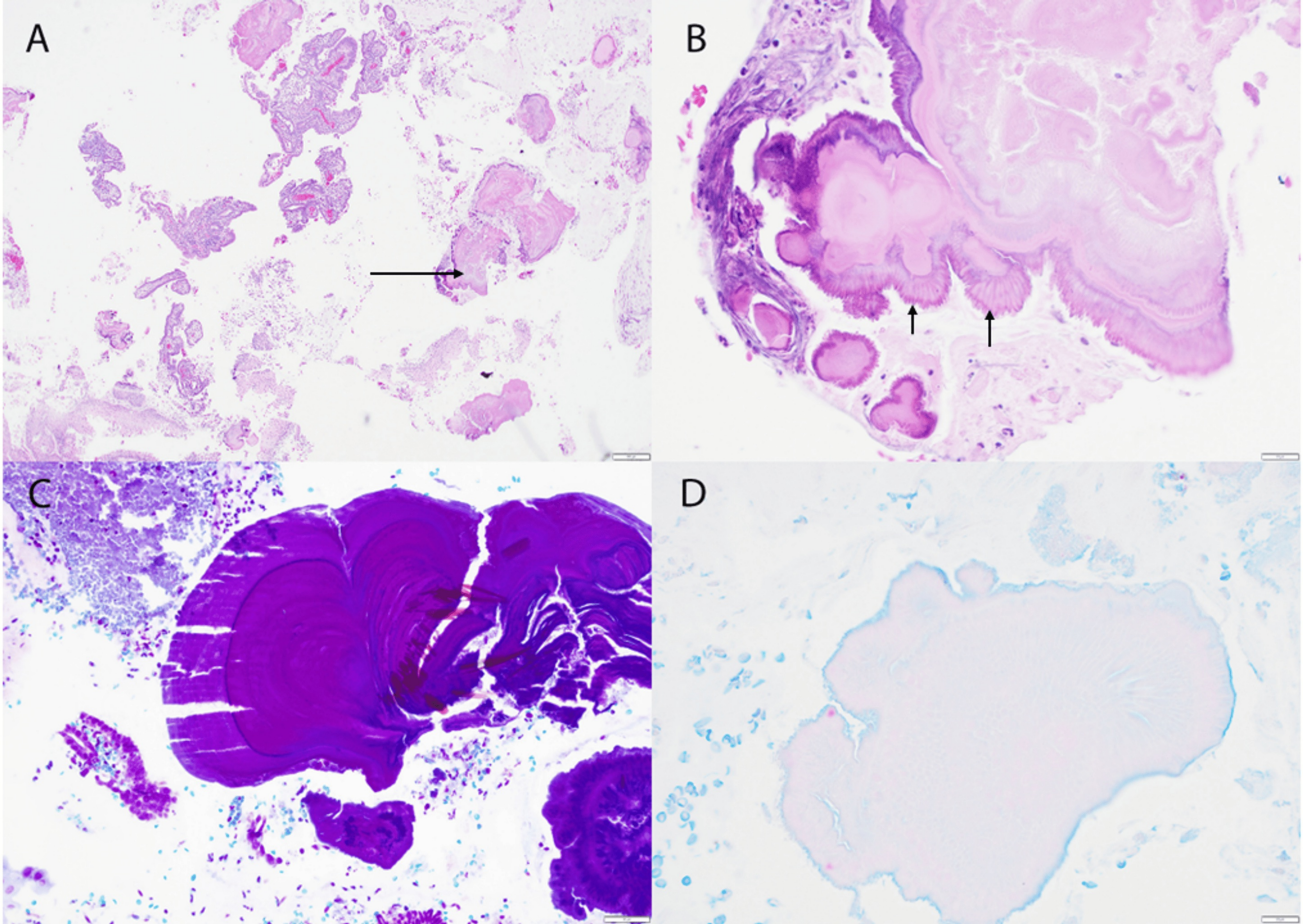
Pane A shows amorphous material with a basophilic core (arrow) in an H&E stain at 40X magnification. In pane B, there are radiating thick club-like peripheral projections (arrow) in an H&E stain at 400X magnification. Pane C demonstrates a negative Gram stain at 200X magnification, and pane D depicts a negative modified acid-fast bacilli stain at 400X magnification.

The patient was a 61-year-old menopausal woman with no apparent symptoms who presented to the clinic for routine gynecological evaluation. A liquid-based pap smear revealed epithelial cell abnormalities with rare, atypical squamous cells of undetermined significance and inflammation. Reflex human papillomavirus (HPV) testing was negative. An ultrasound evaluation of the uterus showed an IUD in place, and an endometrial biopsy was performed. Microscopic evaluation of the endometrial biopsy specimen revealed inactive endometrium with foci of basophilic spherical core and radiating thick club-like peripheral projections (Figures 1A-1B) and detached fragments of non-keratinizing squamous epithelium. Periodic acid-Schiff-diastase (PASD), Gram stain (Figure 1C), and modified acid-fast bacilli (AFB) stains (Figure 1D) were negative. A diagnosis of PAMRAGs with inactive endometrium was made.

## Discussion

O’Brien et al., in their study on endometrial curetting examined during IUD removal, first described PAMRAGs [5]. They occur in the female genital tract, especially in the endometrium. Although PAMRAGs are more common than AMGs, they can coexist in patients with IUDs. Pseudoactinomycotic radiate granules, which can occur in both the presence and absence of IUDs, have been described in the literature as originating from leukocytes aggregating in response to commonly present microorganisms or inert foreign bodies such as IUDs [2, 5]. These studies showed that they contain neutral glycoproteins, lipids, and calcium and are devoid of demonstrable microorganisms, immunoglobulin, complement, or fibrin [2]. There have been contrasting studies on the content of PAMRAGs, making the etiology of PAMRAGs unclear and variable [5-7]. On the other hand, actinomycotic granules are caused by *Actinomyces *species such as *Actinomyces israelii*, a commensal in the genitourinary tract. The colonization of the female genital tract with *Actinomyces *is closely related to the presence of an IUD in the uterine cavity, with an average period of 24-122 months [3].

In histological analysis, peripheral projections of PAMRAGs are typically thick and club-like, lacking any eosinophilic core. Our case report findings align with this description. Pseudoactinomycotic radiate granules may stain nonspecifically with Grocott methenamine silver (GMS) stains. However, AMGs demonstrate yellow-tan granules macroscopically, also called sulfur granules, comprising colonies of actinomyces and inflammatory tissue debris [8]. They exhibit thin, basophilic filaments that radiate from a dense eosinophilic core, and they have extensive inflammatory responses with plasma cells and neutrophils. The GMS stain is positive in AMGs and helpful in highlighting the organisms; AFB stains are negative and distinguish from tuberculosis or *Norcadia *infection [9].

## Conclusions

Pseudoactinomycotic radiate granules can be seen in both IUD and non-IUD users and are more frequent than AMG. They are devoid of bacteria and considered non-pathogenic. In contrast, AMG is seen in long-term IUD use and is associated with tubo-ovarian abscess and PID, thus requiring antibacterial treatment and IUD removal. A finding of PAMRAGs does not preclude AMG presence in the sample. It so warrants a thorough examination of the specimen with knowledge of staining properties, which will aid in making an accurate diagnosis.
